# Stress Is a Risk Factor for Shingles Among Older Adults

**DOI:** 10.1093/geroni/igaa057.1283

**Published:** 2020-12-16

**Authors:** Hyewon Kang, Eileen Crimmins, Jennifer Ailshire

**Affiliations:** University of Southern California, Los Angeles, California, United States

## Abstract

Stress is a risk factor for shingles. Empirical evidence of how stress affects getting shingles is lacking for the older population. This paper examines how chronic stress and stressful events are associated with incident shingles in a nationally representative sample of the population over age 50, the Health and Retirement Study. Using data for 2010-2016, we tracked 12,628 persons aged 50 and older with no history of shingles at 2010 until the first shingles occurrence and linked shingles to chronic stress appraisal and stressful events in the prior period. Chronic stress appraisal was measured in eight life domains: health, spousal/children, finance, work, residence, relationship, alcohol/drug, and caregiving. Adverse life events including spousal loss, involuntary job loss, residential move, negative wealth shock, and spousal onset of disability were included in an index of stressful events. 3.3% of sample members developed new shingles cases. Regression results suggest that having a higher burden of stressful events significantly increased the risk for shingles (OR:1.13, 95% CI=1.05, 1.22), whereas ongoing chronic appraisal was not associated with shingles onset (OR:0.99, 95% CI=0.96, 1.03). Our findings highlight the importance of preventive efforts on stress management in reducing risks for zoster.

